# Progress Toward Rubella and Congenital Rubella Syndrome Elimination — Worldwide, 2012–2024

**DOI:** 10.15585/mmwr.mm7528a1

**Published:** 2026-07-23

**Authors:** Melissa Dahlke, Kimberly A. Dautel, Emilia Vynnycky, Christina Still, Timoleon Papadopoulos, Hyacinthe J. Kabore, Tushar Singh, Laura A. Zimmerman, Michelle Morales

**Affiliations:** ^1^Global Immunization Division, Global Health Center, CDC; ^2^Statistics, Modelling and Economics Department, United Kingdom Health Security Agency, London, United Kingdom; ^3^Department of Infectious Disease Epidemiology and Dynamics, London School of Hygiene & Tropical Medicine, London, United Kingdom; ^4^Division of Viral Diseases, National Center for Immunization and Respiratory Diseases, CDC.

**Summary**

**What is already known about this topic?**

Rubella virus infection during early pregnancy can cause miscarriage, fetal death, stillbirth, or a constellation of birth defects known as congenital rubella syndrome (CRS). Global rubella elimination is achievable.

**What is added by this report?**

During 2012–2024, estimated global coverage with a rubella-containing vaccine (RCV) among infants aged 12–23 months increased from 39% to 73%, and by 2024, 179 of 194 countries had introduced RCV into the routine immunization schedule. During the same period, the number of countries that had achieved rubella elimination verification increased to 117 (60%), reflecting substantial progress in RCV introduction and vaccination coverage. CRS modeling estimates suggest that annual global CRS cases declined by more than 70%, from approximately 101,000 cases in 2012, to 27,000 cases in 2024.

**What are the implications for public health practice?**

Substantial global progress toward rubella and CRS elimination has been made. Through 2024, RCV has been introduced in 179 of 194 countries; however, universal introduction of RCV in all 15 remaining countries and continued strengthening of immunization and surveillance systems are needed to achieve elimination.

## Abstract

Rubella is the leading cause of vaccine-preventable birth defects. Rubella virus infection during pregnancy can result in miscarriage, fetal death, stillbirth, or a constellation of birth defects known as congenital rubella syndrome (CRS). Vaccination is the primary method for preventing rubella and CRS, and a single dose of rubella-containing vaccine (RCV) provides lifelong protection. In 2024, the World Health Organization recommended introduction of RCV in all countries. Achieving rubella elimination is a priority of the Immunization Agenda 2030. This report summarizes global progress toward elimination of rubella and CRS during 2012–2024. During this period, the number of countries that introduced RCV increased from 132 of 194 (68%) to 179 (92%), and global coverage with the first routinely scheduled dose of RCV rose from 39% to 73%. The number of reported rubella cases declined by 66%, from 94,277 in 2012 to 31,593 in 2024, with large outbreaks increasingly concentrated in fewer countries and geographic shifts observed as vaccine introductions advanced. Modeled estimates suggest that CRS cases declined by approximately 70% during the same period. Since 2012 when accelerated rubella control activities began, 117 of 194 (60%) countries have verified elimination of endemic rubella virus transmission. Despite this, in 2024, approximately 21 million infants aged 12–23 months lived in the 15 countries that had not introduced RCV. Although substantial progress has been made, achieving and sustaining rubella and CRS elimination will require RCV introduction in all the remaining countries and continued commitment from all countries to strengthen immunization and surveillance systems.

## Introduction

Rubella is a febrile rash illness and is the leading cause of vaccine-preventable birth defects worldwide (*1*). Although rubella often manifests as a mild illness in children and adults, infection with rubella virus during pregnancy, especially during the first trimester, can result in miscarriage, fetal death, stillbirth, or a constellation of birth defects known as congenital rubella syndrome (CRS). Vaccination is the most effective means of preventing rubella and CRS. A single dose of rubella-containing vaccine (RCV) provides lifelong immunity and vaccination is the cornerstone of global elimination strategies (*2*).

In 2011, the World Health Organization (WHO) recommended introduction of RCV in countries that had achieved and could sustain high measles vaccination coverage, generally defined as ≥80% coverage with the first dose of a measles vaccine delivered through routine vaccination services or supplementary immunization activities, while establishing CRS surveillance (*2*). This recommendation accelerated rubella vaccine introduction and elimination efforts. In 2024, WHO’s Strategic Advisory Group of Experts on Immunization (SAGE) removed the measles vaccination coverage precondition and recommended that all countries introduce RCV into their routine immunization program. SAGE also reinforced the importance of conducting a wide age-range vaccination campaign at the time of RCV introduction to rapidly reduce population susceptibility and accelerate CRS prevention (*3*).

To guide and sustain these efforts, the Measles and Rubella Strategic Framework 2021–2030 (*4*) was developed under the Immunization Agenda 2030 (*5*) and identified rubella elimination as a core global objective. By 2024, five of the six WHO regions had formally adopted rubella elimination goals (*6*). In 2025, the Eastern Mediterranean Region adopted a regional rubella elimination goal, resulting in the establishment of formal elimination targets in all six WHO regions.* This report updates a previous global assessment of progress toward rubella and CRS elimination through 2022 (*6*) and summarizes progress toward elimination during 2012–2024, with an emphasis on developments since publication of the previous report.

## Methods

### Vaccination Activities

Each year, countries report vaccination data through the electronic Joint Reporting Form (eJRF), including information on immunization schedules and the number of vaccine doses administered through routine services and vaccination campaigns. WHO and UNICEF Estimates of National Immunization Coverage for the first routine dose of RCV^†^ are generated using administrative coverage data (number of doses administered divided by the estimated target population), national coverage estimates (official country-reported estimates), and vaccination coverage surveys. Countries were documented as having introduced RCV if the vaccine was included in the national routine childhood immunization schedule.

In this report, eJRF data on RCV introduction, routine vaccination coverage, rubella surveillance, CRS surveillance, and elimination verification status were reviewed for 2012–2024. Analyses focused on 2012 (the year that accelerated rubella elimination efforts began), 2019 (the last complete pre–COVID-19 pandemic year), and 2024 (the most recent year with complete available data). Developments since publication of the previous report through 2022 (*4*) are highlighted. World Bank country income classifications were used to compare trends in RCV introduction and coverage across country income groups.^§^

### Rubella and CRS Surveillance and Modeled CRS Estimates

Rubella and CRS surveillance data are reported through eJRF using standard case definitions (*7*). In many countries, rubella cases are identified through their measles surveillance systems, because both diseases are characterized by fever and rash. Historical surveillance and vaccination coverage data are subject to retrospective revision because countries update previously submitted reports. Therefore, data included in this report might differ from those published previously. CRS cases are often identified through sentinel surveillance sites located at referral hospitals and subspecialty clinics (e.g., cardiology, ophthalmology, and audiology services) and might not be nationally representative, resulting in underestimation of reported CRS cases (*7*). Because of these limitations, country-level CRS case estimates were derived using a previously described dynamic transmission model. The model incorporates routine and supplementary immunization activity coverage data, age-specific seroprevalence data, fertility rates, and demographic data to estimate rubella transmission and CRS cases and incidence^¶^ (*8*). Regional and global estimates were obtained by summing country-level estimates. The Global Measles and Rubella Laboratory Network (GMRLN) comprises 762 laboratories that conduct measles and rubella case confirmation through serologic and molecular testing. The distribution of rubella virus genotypes was reviewed and summarized by the CDC Rubella Laboratory, one of the GRMLN laboratories. This activity was reviewed by CDC, deemed not research, and conducted consistent with applicable federal law and CDC policy.**

### Progress Toward Elimination

Progress toward regional elimination goals was assessed by the number of countries that had introduced RCV and the number of countries with verified elimination of endemic rubella virus transmission. Interruption of endemic rubella virus transmission is defined as the absence of evidence of ongoing endemic rubella transmission for ≥12 months. Countries are considered to have eliminated rubella after sustaining interruption of endemic transmission for ≥36 months in the setting of adequate surveillance; elimination is verified by an independent regional verification commission (*9*). Data on rubella elimination verification status were obtained from regional verification commission reports and supplemented with interim status updates released before publication of full reports.^††^

## Results

### Vaccination Activities

**FIGURE 1:**
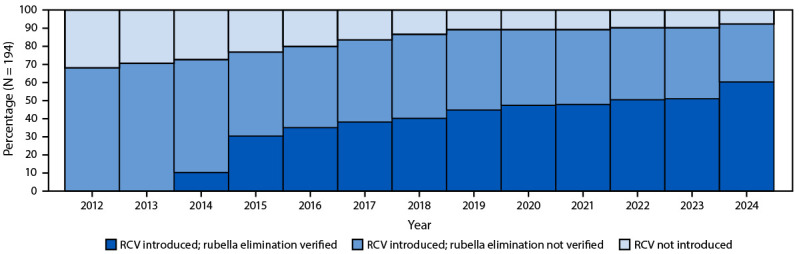
Percentage of countries that introduced rubella-containing vaccine into the routine immunization schedule and with verified rubella elimination, by year — worldwide, 2012–2024 **Abbreviation: **RCV = rubella-containing vaccine.

By 2024, a total of 179 of 194 (92%) countries had introduced RCV,^§§^ a 36% increase compared with the 132 (68%) countries that offered RCV in 2012 (Figure 1). All countries in the Region of the Americas, the European Region, the South-East Asia Region, and the Western Pacific Region have introduced RCV. In the two remaining regions, RCV has been introduced in 35 of 47 (74%) countries in the African Region and 18 of 21 (86%) countries in the Eastern Mediterranean Region (Table).

**TABLE:** Number and percentage of countries with verified rubella elimination and with rubella-containing vaccine in the vaccination schedule, number of rubella and congenital rubella syndrome cases, and estimated congenital rubella syndrome cases, by World Health Organization region — worldwide, 2012, 2019, and 2024

Characteristic	WHO region (no. of countries)						
	AFR n = 47	AMR n = 35	EMR n = 21	EUR n = 53	SEAR n = 10	WPR n = 28	Worldwide N = 194
Regional rubella or CRS target	**Elimination in 80% of countries**	**Regional elimination**	**None**	**Regional elimination**	**Regional elimination**	**Regional elimination**	None
Countries verified eliminated, no. (%)*							
2012	NA	NA	NA	NA	NA	NA	**NA**
2019	NA	35 (100)	NA	45 (85)	NA	4 (15)	**84 (43)**
2024	3 (6)	35 (100)	4 (19)	50 (94)	6 (60)	19 (68)	**117 (60)**
**Countries with RCV in schedule, no. (%)**							
2012	3 (6)	35 (100)	14 (67)	53 (100)	5 (50)	22 (79)	**132 (68)**
2019	31 (66)	35 (100)	16 (76)	53 (100)	10 (100)	28 (100)	**173 (89)**
2024	35 (74)	35 (100)	18 (86)	53 (100)	10 (100)	28 (100)	**179 (92)**
**Regional rubella vaccination coverage (%)^†^**							
2012	0	94	35	95	5	72	**39**
2019	33	87	41	95	94	94	**69**
2024	37	88	73	94	96	90	**73**
**Countries reporting rubella cases, no. (%)**							
2012	41 (87)	35 (100)	19 (90)	47 (89)	10 (100)	24 (86)	**176 (91)**
2019	45 (96)	34 (97)	19 (90)	50 (94)	9 (90)	23 (82)	**180 (93)**
2024	45 (96)	33 (94)	19 (90)	50 (94)	10 (100)	23 (82)	**180 (93)**
**Reported rubella cases, no.**							
2012	10,850	15	1,681	30,579	5,857	45,295	**94,277**
2019	6,026	25	2,603	673	3,824	36,029	**49,180**
2024	24,497	0	2,429	522	2,742	1,403	**31,593**
**Countries reporting CRS cases, no. (%)**							
2012	20 (43)	35 (100)	9 (43)	43 (81)	6 (60)	17 (61)	**130 (67)**
2019	18 (38)	32 (91)	13 (62)	44 (83)	6 (60)	20 (71)	**133 (69)**
2024	20 (43)	31 (89)	17 (81)	46 (87)	8 (80)	18 (64)	**140 (72)**
**Reported CRS cases, no.**							
2012	69	3	20	62	14	134	**302**
2019	9	0	26	9	160	220	**424**
2024	77	1	305	1	14	213	**611**
**Model estimated CRS cases, no. (95% CI)^§^**							
2012	41,319 (20,655–73,276)	3 (<1–417)	6,407 (1,273–15,507)	341 (48–1,690)	46,387 (9,055–95,452)	6,356 (3,567–16,643)	**101,381 (53,527–169,035)**
2019	23,600 (9,067–46,034)	3 (<1–264)	6,456 (978–15,837)	114 (0–1,164)	51 (1–1,451)	4 (1–3,377)	**31,398 (13,204–57,551)**
2024	23,932 (8,934–47,333)	2 (<1–97)	2,224 (381–5,850)	79 (1–829)	<1 (<1–1,154)	3 (<1–578)	**26,859 (11,203–51,429)**

**Abbreviations:** AFR = African Region; AMR = Region of the Americas; CRS = congenital rubella syndrome; EMR = Eastern Mediterranean Region; EUR = European Region; NA = not available; RCV = rubella-containing vaccine; SEAR = South-East Asia Region; WHO = World Health Organization; WPR = Western Pacific Region.

* Established regional verification commissions verify achievement of elimination in six regions (AFR, AMR, EMR, EUR, SEAR, and WPR). Historical values might differ from previously published reports because of updated country submissions and retrospective corrections.

**^†^** RCV coverage estimates are determined by WHO and UNICEF Estimates of National Immunization Coverage.

**^§^** CRS estimates were generated using a previously described transmission dynamic model incorporating vaccination coverage, seroprevalence, demographic, and fertility data. CRS case estimates are the median of 1,000 bootstrap estimates.

In 2012, RCV had been introduced in 11% of 36 low-income countries and 50% of 46 lower–middle-income countries. However, by 2024, RCV introduction had expanded to include 16 of 26 (62%) low-income countries and 46 of 49 (94%) lower–middle-income countries (Supplementary Figure 1).

Global first-dose RCV coverage among infants aged 12–23 months increased from 39% in 2012 to 73% in 2024, with wide regional variation (range = 37% [African Region] to 96% [South-East Asia Region]). In 2024, overall rubella vaccination coverage was 30% in low-income countries, 79% in lower–middle-income countries, 87% in upper–middle-income countries, and 93% in high-income countries. Among countries that had introduced RCV, coverage was 82% in low-income countries, 81% in lower–middle-income countries, 88% in upper–middle-income countries, and 94% in high-income countries.

### Rubella and CRS Surveillance and Modeled CRS Estimates

**FIGURE 2:**
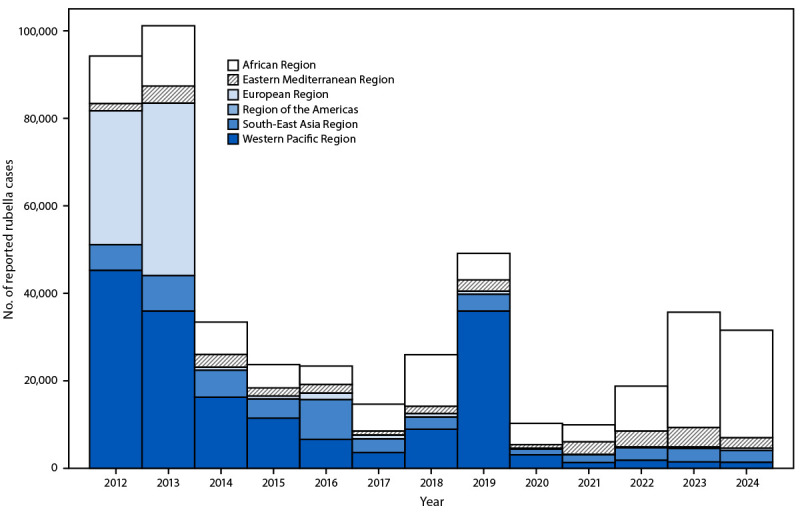
Number of reported rubella cases,* by region and year — worldwide, 2012–2024 * Reported rubella cases in the Region of the Americas were too few to be visible on the scale shown; zero cases were reported in 2022 and 2024, and no more than 25 cases were reported in any other year.

**Reported rubella cases.** The number of countries reporting rubella cases (including the reporting of zero cases) increased from 176 (91%) in 2012 to 180 (93%) in 2024, with a temporary decline to 164 (85%) in 2022 when surveillance and reporting in many countries continued to be affected by the COVID-19 pandemic. The number of reported rubella cases decreased by 80%, from 94,277 in 2012 to 18,881 in 2022, then increased to 31,593 in 2024, with substantial changes in the geographic distribution of reported cases. Whereas most cases in 2012 were reported from the Western Pacific Region and the European Region, by 2024, the African Region accounted for the largest share of reported yearly cases (Figure 2). During 2023 and 2024, two countries^¶¶^ each year accounted for 60% and 67% of global cases, respectively.

**Reported CRS cases.** The number of countries reporting CRS cases increased from 130 (67%) in 2012 to 140 (72%) in 2024. Reported CRS cases increased during the reporting period; 611 CRS cases were reported in 2024, compared with 424 in 2019 and 302 in 2012. Increased case reporting likely reflects, in part, the initiation or expansion of CRS surveillance in several populous countries, including Afghanistan, Bangladesh, India, Indonesia, and Pakistan (Table). Reported CRS cases continued to be detected in countries with recent or incomplete RCV introduction, whereas regions with long-standing RCV use generally reported fewer cases.

**Modeled estimates of CRS cases.** Modeled estimates suggest that the number of CRS cases declined by 73.5% from 2012 through 2024, from 101,381 cases to 26,859 cases (Table). Globally, the modeled estimates of CRS cases per 100,000 live births declined as the percentage of live births in countries that had introduced RCV increased (Supplementary Figure 2). Throughout this period, the African Region accounted for the largest number of estimated CRS cases and, in 2024, represented 89.1% of global estimated CRS cases. Outside of the African Region, CRS cases have been largely concentrated in the Eastern Mediterranean Region since 2019, although the region experienced a 65.6% decline from 2019 to 2024 (Table) (Supplementary Figure 2). By 2024, CRS estimates were lowest in the South-East Asia Region (less than one case), the Region of the Americas (two cases), and the Western Pacific Region (three cases) (Supplementary Figure 3).

**Rubella virus sequence surveillance.** During 2012–2024, rubella viruses from 49 countries were reported to the Rubella Virus Nucleotide Surveillance database. Of the 6,792 viruses sequenced, 56% were genotype 1E and 43% were genotype 2B. As in previous years, most sequences were submitted from China (65%) and Japan (26%); many countries do not send sequences, highlighting persistent gaps in global virologic surveillance.

### Progress Toward Elimination

By 2024, five of the six WHO regions had established rubella and CRS regional elimination goals (*4*). The Region of the Americas was the first region to achieve elimination in 2015, and all 35 countries have maintained elimination status. Verification commissions in the remaining regions assess progress on a country-by-country basis (*8*). The number of countries verified as having eliminated rubella increased steadily from 84 (43%) in 2019 to 117 (60%) in 2024. Elimination status varied by region: three of 47 (6%) in the African Region, all 35 (100%) in the Region of the Americas, four of 21 (19%) in the Eastern Mediterranean Region, 50 of 53 (94%) in the European Region, six of 10 (60%) in the South-East Asia Region, and 19 of 28 (68%) in the Western Pacific Region. Kuwait and Qatar sustained interruption of endemic rubella transmission for >12 months through 2024 but had not yet reached the 36-month period required for verification.

## Discussion

Global progress toward rubella and CRS elimination continues, supported by expanded RCV introduction, ongoing improvements in vaccination and surveillance infrastructure, and sustained leadership commitment. Since accelerated rubella control began in 2012, these investments have enabled 60% of countries to achieve elimination, demonstrating substantial gains in vaccine introduction and childhood vaccination coverage. These advances highlight the effectiveness of global and regional strategies to reduce rubella transmission and prevent CRS and illustrate the feasibility of elimination across diverse epidemiologic and economic settings. Overall, modeled CRS cases declined in parallel with expanded RCV introduction and implementation, demonstrating the effectiveness of global control efforts.

By 2024, approximately 21 million infants aged 12–23 months (16% of the global birth cohort), lived in the 15 countries that had not introduced RCV, primarily in low-income or conflict-affected countries where routine vaccination services face substantial challenges. The 2023–2024 increase in the number of global rubella cases was driven by transmission in Chad and Nigeria, which had not introduced RCV, and in South Africa during RCV rollout, highlighting vulnerability of areas where vaccine access remains incomplete. The introduction of RCV in Nigeria and Democratic Republic of the Congo during 2025–2026 is expected to substantially reduce the number of unvaccinated infants and accelerate progress toward rubella elimination in the African region.

No country that has achieved rubella elimination has experienced reestablished endemic transmission, reflecting the effectiveness of RCV and the importance of maintaining population immunity. As additional countries introduce RCV and strengthen surveillance, progress toward rubella elimination and prevention of CRS are expected to accelerate. However, as long as rubella virus continues to circulate anywhere, the risk for importation remains, including in countries that have achieved elimination. Expanding RCV introduction to all remaining countries is critical to CRS prevention and global elimination efforts (*10*). Strong surveillance, timely outbreak detection, and targeted vaccination of susceptible populations are essential to preventing transmission and closing immunity gaps. Although reported CRS case counts are influenced by surveillance sensitivity and reporting practices, modeled estimates suggest that the global incidence of CRS has declined substantially and will continue to decline as RCV is introduced in the remaining countries.

Accelerated progress will require sustained leadership commitment, continued investment in routine immunization systems, achievement and maintenance of high population immunity through routine vaccination and wide age-range catch-up campaigns at the time of RCV introduction, and targeted strategies to reach unvaccinated and undervaccinated communities. Strategies to reach previously unvaccinated adolescents and adults of childbearing age might also be considered to achieve CRS elimination goals. The 2024 SAGE recommendation removing previous preconditions for RCV introduction provides new opportunities to address remaining immunity gaps (*3*,*10*). With the Eastern Mediterranean Region’s adoption of a regional elimination goal in 2025, all six WHO regions have now established formal targets. Achieving global rubella elimination is feasible but will require RCV introduction in the remaining countries and continued commitment to maintain robust immunization and disease surveillance systems.

### Limitations

The findings in this report are subject to at least three limitations. First, the accuracy and reliability of surveillance and immunization data vary across countries, limiting the ability to identify immunity gaps, guide program strengthening, and verify interruption of transmission. Second, reduced reporting and diminished surveillance quality during the COVID-19 pandemic hindered assessment of progress during that period, and some countries have not returned to prepandemic levels of immunization and surveillance performance. Finally, modeled CRS estimates are subject to limitations, including incomplete availability and representativeness of seroprevalence data in some countries and assumptions that second-dose vaccination is independent of first-dose receipt (*8*).

### Implications for Public Health Practice

Global progress toward rubella and CRS elimination demonstrates the impact of sustained commitment to RCV introduction, strong routine vaccination programs, and timely outbreak detection. Ensuring that all countries introduce RCV and conduct wide age-range catch-up campaigns at the time of RCV introduction remains essential to rapidly reducing susceptibility, preventing CRS, and closing remaining immunity gaps. Strengthening rubella and CRS surveillance, including case detection and virologic data reporting can help identify ongoing transmission and guide focused vaccination activities, particularly in settings with unvaccinated and undervaccinated populations.

The recent SAGE policy update removing previous preconditions for RCV introduction and the adoption of rubella elimination goals across all six WHO regions provide momentum for global action (*3*,*10*). Ongoing RCV introductions in large, high-incidence countries demonstrate that continued expansion of RCV use is feasible even in resource-constrained settings. Continued leadership commitment and investment in immunization and surveillance systems will be critical to sustaining progress and achieving global rubella and CRS elimination.

## Footnotes

*
Eastern Mediterranean countries reaffirm their commitment to health for all at the WHO Regional Committee. Cairo, Egypt: World Health Organization Regional Office for the Eastern Mediterranean; 2025

† Calculated for RCV1, among infants aged 12–23 months or, if RCV1 is given at age ≥1 year, among children aged 24 months. Immunization Analysis and Insights

§ Country income classifications correspond to the World Bank classification for the year analyzed and are updated annually based on gross national income per capita. World Bank Country and Lending Groups – World Bank Data Help Desk

¶ CRS estimates were generated using a transmission dynamic model that incorporates routine and supplementary immunization coverage, seroprevalence, demographic, and fertility data and were updated using World Population Prospects 2024 demographic estimates.

** 45 C.F.R. part 46.102(l)(2), 21 C.F.R. part 56; 42 U.S.C. Sect. 241(d); 5 U.S.C. Sect. 552a; 44 U.S.C. Sect. 3501 et seq.

†† Eastern Mediterranean Region Measles and Rubella Bulletin Jan–June 2025; Measles and Rubella Dashboard, Eastern Mediterranean Region; Eleventh meeting of the European Regional Verification Commission for Measles and Rubella Elimination, 8–10 November 2022; 14th meeting of the European Regional Verification Commission for Measles and Rubella Elimination; Tenth Meeting of the WHO South-East Asia Regional Verification Commission for Measles and Rubella Elimination 22–24 July 2025; Rubella elimination verified in Japan, and measles and rubella elimination verified in Pacific island countries and areas; Fifth annual meeting of the Measles and Rubella Elimination Regional Monitoring and Re-Verification Commission. Mexico City, 4–7 November 2025; and Cabo Verde, Mauritius and Seychelles eliminate measles and rubella | WHO | Regional Office for Africa

§§ The 15 countries that have not introduced RCV as of 2024 are Afghanistan, Central African Republic, Chad, Democratic Republic of the Congo, Djibouti, Equatorial Guinea, Ethiopia, Gabon, Guinea, Liberia, Madagascar, Niger, Nigeria, Somalia, and South Sudan. In the modeled estimates, because of the availability of vaccination coverage data, vaccination in South Africa was not included, and Sudan is assumed to have introduced RCV after a supplementary immunization activity in 2023.

¶¶ In 2023: Chad (RCV not introduced) = 10,918 cases and Nigeria (RCV not introduced) = 10,221 cases. In 2024: Chad (RCV not introduced) = 6,177 cases and South Africa (RCV introduced during 2024) = 15,100 cases.
